# Lenalidomide downregulates ACE2 protein abundance to alleviate infection by SARS-CoV-2 spike protein conditioned pseudoviruses

**DOI:** 10.1038/s41392-021-00608-1

**Published:** 2021-05-10

**Authors:** Siyuan Su, Jianfeng Chen, Ying Wang, Lilly M. Wong, Zhichuan Zhu, Guochun Jiang, Pengda Liu

**Affiliations:** 1 Lineberger Comprehensive Cancer Center, The University of North Carolina at Chapel Hill, Chapel Hill, NC USA; 2 Department of Biochemistry and Biophysics, The University of North Carolina at Chapel Hill, Chapel Hill, NC USA; 3 UNC HIV Cure Center, Institute of Global Health and Infectious Diseases, The University of North Carolina at Chapel Hill, Chapel Hill, NC USA

**Keywords:** Biochemistry, Cell biology

**Dear Editor**,

The recent health emergency caused by SARS-CoV-2 created a global pandemic. Similar to other CoVs, SARS-CoV-2 utilizes its Spike (S) protein to specifically recognize the human angiotensin converting enzyme 2 (ACE2) membrane receptor for infection. Thus, blocking SARS-CoV-2-S/ACE2 interactions has been investigated as a therapeutic direction in treating SARS-CoV-2, including recombinant hACE2 proteins, ACE2-derived peptides, neutralizing antibodies, engineered ACE2 traps, various heparins, TMPRSS2 inhibitors, and others.^1^ Deep mutagenesis assays identified engineered ACE2 mutants with enhanced binding affinity with SARS-CoV-2-S proteins, facilitating development of approaches targeting SARS-CoV-2 S protein binding to ACE2. ACE2 is a plasma membrane attached enzyme converting angiotensin II into angiotensin (1–7) that lowers blood pressure. ACE2 is broadly expressed but detectable expression is enriched in alveolar cells, enterocytes, arterial, and venous endothelial cells. The E3 ligase MDM2 was reported to target ACE2 for degradation in an AMPK phosphorylation-dependent manner.^2^ Intriguingly, increased ACE2 levels were observed in lungs from patients with comorbidities associated with severe COVID-19.^3^ Modulating ACE2 protein homeostasis is an attractive direction to prevent/control SARS-CoV-2 infection; however, regulatory mechanisms of ACE2 protein control remain elusive.

We profiled ACE2 protein expression in a panel of commonly used cell lines, and only observed a detectable ACE2 signal (~130 KD) in UMRC2 cells using an antibody targeting ACE2 C-terminus (sc-390851) (Supplementary Fig. 1a–c). This signal was also detected by an ACE2 N-terminus targeting antibody (CST#4355), with an additional band at ~75 KD, which may correspond to a shorter isoform of human-ACE2 (uniport Q9BYF1). We further examined additional kidney cancer cells and observed full-length ACE2 in UMRC2, UMRC6, and RCC4 cells, with the lower 75 KD band (Fig. 1a). Depleting endogenous ACE2 by shRNAs in UMRC2, UMRC6, or A498 cells led to reduced intensity of the upper 130 KD band with minimal effects on the lower 75KD band (Supplementary Fig. 1d–f), suggesting only the ~130 KD ACE2 band is ACE2. This was further confirmed by PCR analyses of cDNAs obtained from these kidney lines that full-length ACE2 genes were observed in UMRC2, UMRC6, and RCC4 cells (Supplementary Fig. 1g). Thus, we use these three cell lines to study ACE2 protein homeostasis, and the ACE2-C antibody to monitor full-length ACE2 proteins. Depleting ACE2 in either UMRC2 or UMRC6 cells didn’t significantly affect cell growth in vitro (Supplementary Fig. 1h–k).

**Fig. 1:**
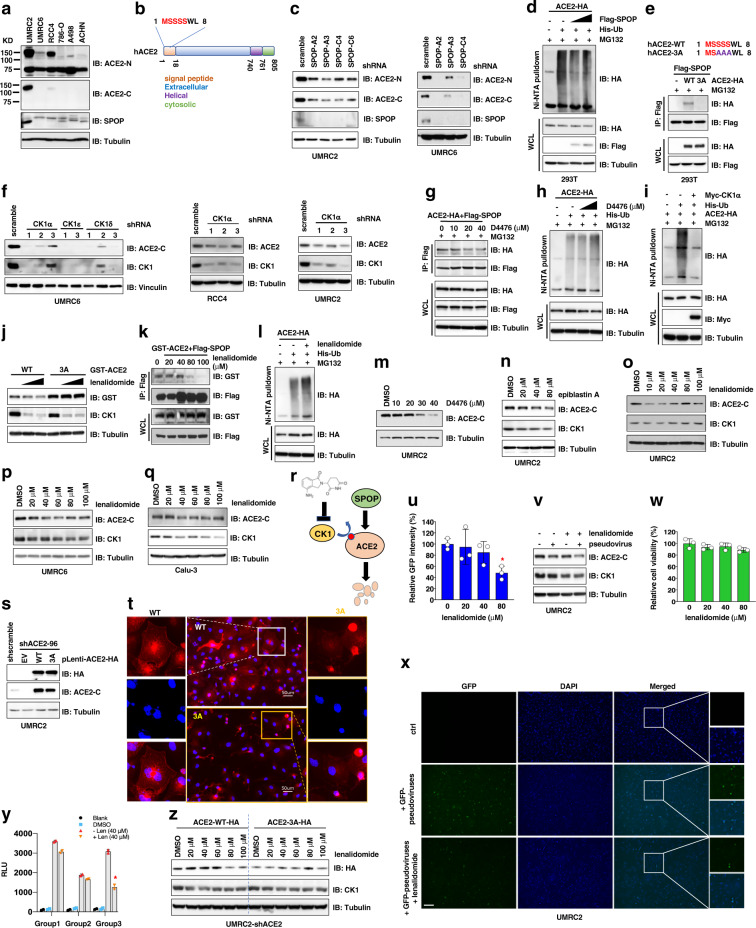
Inactivation of SPOP/CK1 signaling destabilizes ACE2 proteins to alleviate SARS-CoV-2 S protein conditioned pseudoviral infection. **a** Immunoblot (IB) analysis of whole cell lysates (WCL) derived from indicated cell lines. **b** A cartoon illustration to demonstrate that the putative SPOP degron is located within the first five amino acids in the ACE2 signal peptide motif. **c** IB analyses of WCL derived from indicated cells depleted of endogenous SPOP by lenti-viral shRNAs. Cells were selected with 1 μg/ml puromycin for 72 h to eliminate non-infected cells before cell collection. **d** IB analyses of WCL and Ni-NTA pulldowns derived from HEK293T cells transfected with indicated DNA constructs. 10 μM MG132 was added to cell culture 10 h prior to cell collection. **e** Top, an illustration of the ACE2 mutation and deletion generated in this panel. Bottom, IB analyses of WCL and Flag-IPs derived from HEK293T cells transfected with indicated DNA constructs. **f** IB analyses of WCL derived from UMRC6, RCC4, and UMRC2 cells depleted of indicated endogenous CK1 isoforms by lenti-viral shRNAs. Cells were selected with 1 μg/ml puromycin for 72 h to eliminate non-infected cells before cell collection. **g** IB analyses of WCL and Flag-IPs derived from HEK293T cells transfected with pLenti-ACE2-HA and Flag-SPOP. Indicated doses of D4476 and 10 μM MG132 was added to cell culture 10 h prior to cell collection. **h** IB analyses of WCL and Ni-NTA pulldowns derived from HEK293T cells transfected with indicated DNA constructs. 20 or 40 μM D4476 with 10 μM MG132 were added to cell culture 10 h prior to cell collection. **i** IB analyses of WCL and Ni-NTA pulldowns derived from HEK293T cells transfected with indicated DNA constructs. 10 μM MG132 was added to cell culture 10 h prior to cell collection. **j** IB analysis of WCL derived from HEK293T cells transfected with indicated CMV-GST-ACE2 constructs. 3 or 30 μM lenalidomide ﻿was added to cell culture 10 h prior to cell collection. **k** IB analyses of WCL and Flag-IPs derived from HEK293T cells transfected with CMV-GST-ACE2 and Flag-SPOP. Indicated doses of lenalidomide was added to cell culture 10 h prior to cell collection. **l** IB analyses of WCL and Ni-NTA pulldowns derived from HEK293T cells transfected with indicated DNA constructs. 40 μM lenalidomide and 10 μM MG132 ﻿were added to cell culture 10 h prior to cell collection. **m**, **n** IB analyses of WCL derived from UMRC2 cells treated with indicated doses of CK1 inhibitor D4476 (**m**) or epiblastin A (**n**) for 10 h. **o**–**q** IB analyses of WCL derived from UMRC2 (**o**), UMRC6 (**p**), and Calu-3 (**q**) cells treated with indicated doses of CK1α PROTAC lenalidomide for 10 h. **r** A cartoon illustration of a proposed model that lenalidomide targets CK1 for CRBN-mediated degradation, alleviating SPOP binding and protection on ACE2 proteins, therefore leading to reduced ACE2 protein abundance. **s** IB analyses of WCL derived from endogenous ACE2-depleted UMRC2 cells infected with indicated pLenti-ACE2-HA viruses. **t** Representative immunofluorescent images using WT- and 3A-ACE2 UMRC2 cells obtained from (**s**) immune-stained with an HA antibody to indicate ACE2 signals. **u** Pretreating UMRC2 cells with 80 μM lenalidomide for 24 h reduced infection by GFP expressing SARS-CoV-2 S protein conditioned pseudoviruses in vitro. GFP signals were measured 24 h post-infection. Please refer to “Methods” section for details. Error bars were calculated as mean +/− SD, *n* = 3. *p < 0.05 (one-way *ANOVA* test). **v** IB analyses of WCL derived from UMRC2 cells with indicated treatments. Where indicated, 40 μM lenalidomide was added to cell culture 7 h prior to cell collection. **w** GFP expressing SARS-CoV-2 S protein conditioned pseudovirus infection and lenalidomide treatment do not significantly affect cell proliferation in vitro determined by CellTiter-Glo assays. Error bars were calculated as mean +/− SD, *n* = 3. *p < 0.05 (one-way *ANOVA* test). **x** Representative immunofluorescent images indicating that lenalidomide pre-treatment reduces infection of UMRC2 cells by GFP expressing SARS-CoV-2 S protein conditioned pseudoviruses in vitro. The bar indicates 50 μm. **y** Pre-treatment of UMRC2 cells with lenalidomide reduces infection by home-made luciferase-expressing SARS-CoV-2 S protein conditioned pseudoviruses in vitro. UMRC2 cells were treated with 40 μM lenalidomide for 7 h before pseudoviral infection in all groups. Group 1, UMRC2 cells were infected with 500 μL home-made pseudoviruses then treated with 40 μM lenalidomide immediately after spinoculation. Group 2, UMRC2 cells were infected with 500 μL home-made pseudoviruses then treated with 40 μM lenalidomide immediately after spinoculation, followed by refreshing culture with 40 μM lenalidomide 24 h post-spinoculation. Group 3, UMRC2 cells were infected with 500 μL home-made pseudoviruses then treated with 40 μM lenalidomide immediately after spinoculation, followed by refreshing culture with 40 μM lenalidomide 24 and 48 h post-spinoculation, respectively. Viral infection was monitored by RLU (relative light unit) generated in luciferase activity assays. Error bars were calculated as mean +/− SD, *n* = 2. *p < 0.05 (one-way *ANOVA* test). **z** IB analyses of WCL derived from indicated ACE2 expressing UMRC2 cells treated with indicated doses of lenalidomide for 20 h prior to cell collection

ACE2 protein sequence includes a putative degron for the E3 ligase SPOP, defined as _1_-MSSSS-_5_, located at the extreme N-terminus of ACE2 signal peptide (amino acid 1–17) (Fig. 1b and Supplementary Fig. 2a). SPOP depletion reduced ACE2 protein abundance in UMRC2, UMRC6 (Fig. 1c) and RCC4 cells (Supplementary Fig. 2b), and didn’t affect UMRC6 cell growth in vitro (Supplementary Fig. 2c), nor constantly downregulating ACE2 mRNAs (Supplementary Fig. 2d, e). Ectopic SPOP expression reduced ubiquitination of either C-terminal HA-tagged ACE2 (Fig. 1d) or N-terminal GST-tagged ACE2 (Supplementary Fig. 3a), suggesting SPOP may interfere with ACE2 ubiquitination.

Mutating SPOP degron _1_-MSSSS-_5_ into _1_-MSAAA-_5_ (3A), or deleting _3_-SSS-_5_ reduced ACE2 binding to SPOP (Fig. 1e and Supplementary Fig. 3b), supporting _1_-MSSSS-_5_ as a major motif for SPOP recognition. Given casein kinase(s) phosphorylates SPOP degrons to modulate SPOP binding, depletion of endogenous CK1α reduced ACE2 protein abundance in UMRC6, RCC4, and UMRC2 cells (Fig. 1f). CK1α depletion-induced ACE2 downregulation was partially rescued by MG132 (Supplementary Fig. 3c). Inhibiting CK1 by D4476 reduced ACE2 binding to SPOP and increased ACE2 ubiquitination (Fig. 1g, h and Supplementary Fig. 3d, e). D4476 treatment-induced ACE2 downregulation was observed in WT but not 3A-ACE2 expressing cells (Supplementary Fig. 3f), supporting _3_-SSS-_5_ as major CK1 phosphorylation sites. CK1α expression reduced ACE2 ubiquitination (Fig. 1i). Intriguingly, lenalidomide, an FDA approved drug in treating multiple myeloma, follicular and marginal zone lymphoma, as a CK1α PROTAC,^4^ efficiently induced degradation of WT but not 3A-ACE2 (Fig. 1j) and attenuated ACE2 binding to SPOP (Fig. 1k), resulting in enhanced ACE2 ubiquitination (Fig. 1l and Supplementary Fig. 3g). These data support CK1-mediated ACE2-Ser3/Ser4/Ser5 phosphorylation triggering ACE2 recognition by SPOP.

Next, we examined if inactivating CK1 affects endogenous ACE2 abundance. Inhibiting CK1 by D4476 or epiblastin A reduced ACE2 proteins in UMRC2 (Fig. 1m, n), UMRC6 (Supplementary Fig. 4a, b) and RCC4 (Supplementary Fig. 4c, d) cells. Lenalidomide as a CK1α PROTAC led to a canonical biphasic response of ACE2 expression in UMRC2, UMRC6, RCC4, and Calu-3 (Fig. 1o–q, and Supplementary Fig. 4e, f) cells. Lenalidomide-like compounds including CC-122, pomalidomide, and thalidomide^4^ didn’t reduce CK1 expression nor ACE2 protein abundance (Supplementary Fig. 4g–i). These data suggest CK1 inactivation reduces ACE2 protein abundance (Fig. 1r).

Given NEDD4 and MDM2^2^ have been reported to be involved in ACE2 homeostasis control, we found ectopic expression of NEDD4 or MDM2 reduced ACE2 expression (Supplementary Fig. 5a, b). However, depleting neither NEDD4 nor MDM2 by sgRNAs upregulated ACE2 proteins in UMRC2 and UMRC6 cells (Supplementary Fig. 5c–h), suggesting NEDD4 and MDM2 may not be physiological ACE2 degrading E3 ligases in this setting. Thus, if SPOP/CK1 protects ACE2 by competing with ACE2 degrading E3 ligase(s) remains to be determined. Given the SPOP degron presenting in ACE2 N-terminal signal peptide is essential for ACE2 plasma membrane localization, SPOP depletion seemed to reduce ACE2 plasma membrane localization (Supplementary Fig. 6a, b). Compared with WT-, 3A-ACE2 was deficient in plasma membrane enrichment (Fig. 1s–t and Supplementary Fig. 6c), suggesting CK1/SPOP binding may occur in cytoplasm to facilitate ACE2 trafficking to plasma membrane that warrants more in-depth investigations. Inserting a “MTSSSS” sequence or the ACE2 signal peptide sequence (aa 1–17) into N-terminus of a nuclear/cytoplasmic protein cGAS slightly enhanced cGAS binding with SPOP (Supplementary Fig. 7a, b), supporting a possible role of this motif in mediating ACE2 binding with SPOP.

We next tested if lenalidomide blocks SARS-CoV-2 pseudoviral infection. We pretreated UMRC2 cells with various doses of lenalidomide before adding SARS-CoV-2 S protein pseudo-typed GFP expressing baculoviruses, and found 80 μM lenalidomide pre-treatment blocked ~40% viral infection in vitro (Fig. 1u–v, with an IC50 ~168.1 μM, Supplementary Fig. 8a, b), without significantly affecting cell proliferation (Fig. 1w). Decreased pseudo-viral infection in UMRC2 cells was also evidenced by reduced GFP signals (Fig. 1x). Pre-treating UMRC2 cells with 40 μM lenalidomide also efficiently reduced home-made pseudoviral infection (Fig. 1y). Co-administration of lenalidomide in UMRC2 cells simultaneously with pseudovirus also reduced viral infection load (Supplementary Fig. 8c), which was not observed in 3A-ACE2 expressing UMRC2 cells (Fig. 1z and Supplementary Fig. 8d–f). These data support a promise for using lenalidomide in preventing infection/reinfection by SARS-CoV-2 through inactivating SPOP/CK1/ACE2 signaling. However, if lenalidomide-induced ACE2 downregulation affects other ACE2 physiologocal function would need to be considered.

Here, we report an FDA approved drug lenalidomide that may be repurposed to suppress SARS-CoV-2 infection. Given that the current studies focus on the in vitro pseudo-viral infection model, lenalidomide will need to be examined in animal models to test its efficacy in blocking SARS-CoV-2 infection and effects on normal ACE2 physiological function, that observed in mouse vascular smooth muscle cells but not UMRC2 cells (Supplementary Fig. 9a–f). Recently AXL,^5^ ASGR1 and KREN1 were also reported as additional host receptors for SARS-CoV-2 S protein (bioRxiv). Interestingly, like ACE2, both ASGR1 and KREN1 contain putative SPOP degrons (Supplementary Fig. 9g). Thus, SPOP/CK1 signaling may also control ASGR1 and KREN1 protein homeostasis, and lenalidomide may exert a broader function in suppressing SARS-CoV-2 infection by functioning through multiple S protein receptors. However, further in-depth investigations are warranted to examine these possibilities.

## Supplementary information

Supplementary Information

## Data Availability

The data used for the current study are available from the corresponding author upon reasonable request.
