# Supplementary material for: Lenalidomide downregulates ACE2 protein abundance to alleviate infection by SARS-CoV-2 spike protein conditioned pseudoviruses
Source: Signal Transduct Target Ther. 2021 May 10;6:182. doi: 10.1038/s41392-021-00608-1 (PMC8107201; doi:10.1038/s41392-021-00608-1)
Supplement: Supplementary file 1 — Supplementary Information [file 41392_2021_608_MOESM1_ESM.pdf]

Supplementary Materials for

Lenalidomide downregulates ACE2 protein abundance to alleviate infection by SARS-CoV-2 spike protein conditioned pseudoviruses

Siyuan Su, Jianfeng Chen, Ying Wang, Lilly M. Wong, Zhichuan Zhu, Guochun Jiang and Pengda Liu

Correspondence to: pengda_liu@med.unc.edu

This PDF file includes:

Materials and Methods
Figures. S1 to S9

Other Supplementary Materials for this manuscript include the following:

None.

Materials and Methods

Cell Culture

HEK293T, HeLa, T98G, BPH1, A673, UMRC2, UMRC6, RCC4, 786-O, A498, MHH-ES-1 and Calu-3 cells were cultured in DMEM supplemented with 10% fetal bovine serum, 100 units of penicillin and 100 mg/ml streptomycin at 37 °C with 5% CO_2. A549, H1299 and H358 cells were cultured in RPMI-1640 supplemented with 10% fetal bovine serum, 100 units of penicillin and 100 mg/ml streptomycin at 37 °C with 5% CO_2. Mouse vascular smooth muscle cells (VSMC) were cultured in F12-DMEM supplemented with 10% fetal bovine serum, 100 units of penicillin and 100 mg/ml streptomycin at 37 ^{o}C with 5% CO_2.

Transfection and Lentiviral Production

Cell transfection was performed using lipofectamine 3000 (Invitrogen) or polyethylenimine (PEI) (Polysciences), as described previously [6-8]. Packaging of lentiviral shRNA expressing viruses, as well as subsequent infection of UMRC2, UMRC6, RCC4 and A498 cells were performed according to the protocols described previously [9-11]. Following viral infection, cells were maintained in the presence of puromycin (1 μg/ml) for 3 days before cell collection.

Plasmids

The pCDNA3.1-hACE2-C9 plasmid was purchased from Addgene (#145033). pCMV-GST-ACE2 was constructed by cloning ACE2 into SalI/NotI sites on pCMV-GST vector. ACE2-3A mutant and ΔSSS mutant were constructed by including mutated sequence in the forward PCR primers as listed below. pLenti-blasticidin-ACE2-WT-HA and pLenti-blasticidin-ACE2-3A-HA were cloned by cloning ACE2-WT and 3A (from pCDNA3.1-hACE2-C9 plasmid using hACE2-AgeI-F or hACE2-3A-AgeI-F and hACE2-SalI-HA-R primers) into pLenti-blasticidin-CMV-GFP plasmid. Flag-SPOP and His-ubiquitin plasmids were constructed in a previous study [12], Flag-NEDD4 was constructed in a previous study [13]. Myc-MDM2 is kindly shared by Yue Xiong lab at UNC-Chapel Hill. MSSSS-cGAS and ACE2-signal-peptide-cGAS were cloned (using MSSSS-cGAS-BglII-F or ACE2-sigpeptide-cGAS-F and hcGAS-XhoI-R) into the pCDNA3.0 vector.

Primers for cloning are listed below:
hACE2-SalI-F: 5’-GCAT GTCGACT ATGTCAAGCTCTTCCTGGCTCC-3’
hACE2-NotI-R: 5’-GCAT GCGGCCGCCTAAAAGGAGGTCTGAACATCATCAGTG-3’
hACE2-3A-SalI-F: 5’-GCAT GTCGACTATGTCAGCCGCTGCC TGGCTCCTTCTCAGCC-3’
hACE2-deleteSSS-SalI-F: 5’-GCATGTCGACT ATGTCA TGGCTCCTTCTCAGCC-3’
hACE2-AgeI-F: 5’-GCAT ACCGGTATGTCAAGCTCTTCCTGGCTCC-3’
hACE2-3A-AgeI-F: 5’-GCAT ACCGGT ATGTCAGCCGCTGCC TGGCTCCTTCTCAGCC-3’
hACE2-SalI-HA-R: 5’-GCAT GTCGACTCTAAGCGTAATCTGGAACATCGTATGGGTAA AAGGAGGTCTGAACA-3’

RT-PCR primers used in this study are listed below:
hSPOP-RT-PCR-F: 5’-GCCCTCTGCAGTAACCTGTC-3’
hSPOP-RT-PCR-R: 5’-GTCTCCAAGACATCCGAAGC-3’
hACE2-RT-PCR-F: 5’-TCCAGGGAACAGGTAGAGGA-3’
hACE2-RT-PCR-R: 5’-GCTCAGCACTGCTCAAACAC-3’
hβ-actin-F: 5’-CCTGGCACCCAGCACAAT-3’

hβ-actin-R: 5’-GCCGATCCACACGGAGTA-3’
Mouse SMA-RT-PCR-F: 5’-CGCTGTCAGGAACCCTGAGA-3’
Mouse SMA-RT-PCR-R: 5’-CGAAGCCGGCCTTACAGAG-3’
Mouse CNN1-RT-PCR-F: 5’-CTCTGCTTTGCCAGAGCCCCC-3’
Mouse CNN1-RT-PCR-R: 5’-TGACGCCGTGTACCGTAGGC-3’
Mouse MHC-RT-PCR-F: 5’-GTTCATTCGCATCAACTTCG-3’
Mouse MHC-RT-PCR-R: 5’-GGCGAGCAGGTAGTAGAAG-3’

shRNAs used in this study were purchased from Sigma and their sequences are listed below:
shACE2-55:
CCGGTTATGCCTCCATCGATATTAGCTCGAGCTAATATCGATGGAGGCATAATTTTTG
shACE2-96:
CCGGGCTGGACAGAAACTGTTCAATCTCGAGATTGAACAGTTTCTGTCCAGCTTTTTG
shACE2-97:
CCGGGCCGAAGACCTGTTCTATCAACTCGAGTTGATAGAACAGGTCTTCGGCTTTTTG
shSPOP-A2:
CCGGCACAGATCAAGGTAGTGAAATCTCGAGATTTCACTACCTTGATCTGTGTTTTTG
shSPOP-A3:
CCGGCAAGGTAGTGAAATTCTCCTACTCGAGTAGGAGAATTTCACTACCTTGTTTTTG
shSPOP-C4:
CCGGCAAACGCCTGAAGCAATCCTACTCGAGTAGGATTGCTTCAGGCGTTTGTTTTTG
shSPOP-C6:
CCGGCTCCTACATGTGGACCATCAACTCGAGTTGATGGTCCACATGTAGGAGTTTTTG
shCK1α -1:
CCGGGCCACAGTTGTGATGGTTGTTCTCGAGAACAACCATCACAACTGTGGCTTTTTG
shCK1α -2:
CCGGGCAAGCTCTATAAGATTCTTCCTCGAGGAAGAATCTTATAGAGCTTGCTTTTTG
shCK1α-3:
CCGGGCAGAATTTGCGATGTACTTACTCGAGTAAGTACATCGCAAATTCTGCTTTTT
shCK1ε -1:
CCGGCCAAGAGACAGAAATACGAAACTCGAGTTTCGTATTTCTGTCTCTTGGTTTTTG
shCK1ε -2:
CCGGCCAAGAGACAGAAATACGAAACTCGAGTTTCGTATTTCTGTCTCTTGGTTTTT
shCK1ε-3:
CCGGCCGATGAGAACTCTCCTTATTCTCGAGAATAAGGAGAGTTCTCATCGGTTTTTG
shCK1δ -1:
CCGGCCAGTGTTTGCTTAGTGTCTTCTCGAGAAGACACTAAGCAAACACTGGTTTTT
shCK1δ -2:
CCGGGTATATCCACTCCAAGAACTTCTCGAGAAGTTCTTGGAGTGGATATACTTTTT
shCK1δ-3:
CCGGCCTGTGTCTACTAACAAGGACCTCGAGGTCCTTGTTAGTAGACACAGGTTTTTG

sgRNA sequence used to deplete endogenous NEDD4 and MDM2 are listed below:
hNEDD4-sg1-F: CACCGTTCGGAAATGGCAACTTGCG
hNEDD4-sg1-R: AAACCGCAAGTTGCCATTTCCGAAC
hNEDD4-sg2-F: CACCGCCAACCGGTAATGGATAAAG
hNEDD4-sg2-R: AAACCTTTATCCATTACCGGTTGGC
hNEDD4-sg3-F: CACCGTGTTCCACTTTATCCATTAC
hNEDD4-sg3-R: AAACGTAATGGATAAAGTGGAACAC

hNEDD4-sg4-F: CACCGGTCCCCGCACCTCGTCCTCC
hNEDD4-sg4-R: AAACGGAGGACGAGGTGCGGGGACC
hMdm2-sg1-F: CACCGAGGGTCTCTTGTTCCGAAGC
hMdm2-sg1-R: AAACGCTTCGGAACAAGAGACCCTC
hMdm2-sg2-F: CACCGATCGTTTAGTCATAATATAC
hMdm2-sg2-R: AAACGTATATTATGACTAAACGATC
hMdm2-sg3-F: CACCGGTGGTTACAGCACCATCAGT
hMdm2-sg3-R: AAACACTGATGGTGCTGTAACCACC
hMdm2-sg4-F: CACCGCTTGGTAGTAGTCAATCAGC
hMdm2-sg4-R: AAACGCTGATTGACTACTACCAAGC

Primers to generate shACE2-#49 resistance ACE2 is listed below:
hACE2-sh49-resistant-F: 5'-
CAAACTCTACAGAAGCTGGACAAAAGCTATTTAATATGCTGAGGCTTGG-3'
hACE2-sh49-resistant-R: 5'-
CCAAGCCTCAGCATATTAAATAGCTTTTGTCCAGCTTCTGTAGAGTTTG-3'

Primers to generate MSSSS-cGAS, or ACE2-signal-peptide-cGAS constructs are listed below:
MSSSS-cGAS-BglII-F: 5'-
GCATAGATCTATGTCAAGCTCTTCCCAGCCTTGGCACGGAAAGGC-3'
ACE2-sigpeptide-cGAS-BglII-F: 5'-
GCATAGATCTATGTCAAGCTCTTCCTGGCTCCTTCTCAGCCTTGTTGCTGTAACTGCT
GCTCAG CAGCCTTGGCACGGAAAGGC-3'
hcGAS-XhoI-R: 5'-GCATCTCGAG TCAAAATTCATCAAAAACTGG-3'
3xHA-hcGAS-SalI-R: 5'-GCAGTCGACTACCCCTACGACGTGCCCGACTACGCCGGCTAT
CCGTATGATGTCCCGGACTAGCAGGATCCTCAAAATTCATCAAAAACTGGAAACTCA
-3'

PCR validation of ACE2 isoforms are listed below:
ACE2- N-F1: 5'-CCTGGCTCCTTCTCAGCC-3' (nt: 5)
ACE2- N-R1: 5'-CCTCAGATCTCCAGCTTTCCC-3' (nt: 500)
ACE2- N-F2: 5'-GAACATCTTCATGCCTATGTGAGG-3' (nt: 710)
ACE2- N-R2: 5'-CGTGAGTGCTTGTTTGAGCAGG-3' (nt: 1335)
ACE2-break-F3: 5'-GGCCCTCTGCACAAATGTGACATC-3' (nt: 1609)
ACE2-break-R3: 5'-CTGTTGTCATTCAGACGGAAAGCATC-3' (nt: 2162)
ACE2-C-F4: 5'-CCTGGCTGAAAGACCAGAACAAG-3' (nt: 1778)
ACE2-C-R4: 5'-CACTATCACTCCCATCACAACTCC-3' (nt: 2262)

Immunoblot and Immunoprecipitations Analyses

Cells were lysed in EBC buffer (50 mM Tris pH 7.5, 120 mM NaCl, 0.5% NP-40) supplemented with protease inhibitors (Complete Mini, Bimake) and phosphatase inhibitors (phosphatase inhibitor cocktail set I and II, Bimake). The protein concentrations of whole cell lysates were measured by NanoDrop OneC (Thermo Scientific) using the Bio-Rad protein assay reagent as described previously [6]. Equal amounts of whole cell lysates were resolved by SDS-PAGE and immunoblotted with indicated antibodies. For immunoprecipitations analysis, 1000 μg lysates containing tagged molecules were incubated with 10 μL agarose beads coupled antibodies for the

specific tag for 4 hr at 4 °C. The recovered immuno-complexes were washed five times with NETN buffer (20 mM Tris, pH 8.0, 100 mM NaCl, 1 mM EDTA and 0.5% NP-40) before being resolved by SDS-PAGE and immunoblotted with indicated antibodies.

Key reagents table

REAGENT or RESOURCE	SOURCE	IDENTIFIER
**Beads and Recombinant Proteins**		
Anti-HA agarose beads	Sigma	cat#A-2095, lot#057M4864V
Anti-Flag agarose beads	Sigma	cat#A2220, lot#SLBW1929
Ni-NTA agarose beads	Goldbio	Cat#H-350-100, lot# 1086.021919A
**Antibodies**		
Anti-ACE2-N antibody	Cell Signaling Technology	cat#4355, lot#1
Anti-ACE2-C antibody	Santa Cruz Biotechnology	sc-390851, lot#D2420
Anti-TMPRSS2 antibody	Santa Cruz Biotechnology	sc-515727, lot#D2720
Anti-SPOP antibody	Proteintech	16750-1-AP, lot#00024790
Anti-CK1 antibody	Cell Signaling Technology	cat#2655, lot#2
Anti-HA antibody	Cell Signaling Technology	cat#3724, lot#8
Anti-Flag antibody (Flag-M2 antibody)	Sigma-Aldrich	cat#F1804, lot#SLBN8915V
Anti-GST antibody	Santa Cruz Biotechnology	sc-459, lot#S1515
Anti-Myc tag antibody	Proteintech	cat#60003-2-Ig
Anti-MDM2 antibody	Cell Signaling Technology	cat#86934, lot#2
Anti-NEDD4 antibody	Cell Signaling Technology	cat#2740, lot#2
Anti-phospho-ERK-p42/p44 antibody	Cell Signaling Technology	cat#9101
Anti-total ERK1 antibody	Santa Cruz Biotechnology	sc-271269, lot#F1917
Anti-cMyc antibody	Cell Signaling Technology	cat#83623, lot#1
Anti-cGAS antibody	Cell Signaling Technology	cat#18583, lot#1
Anti-Akt-pS473 antibody	Cell Signaling Technology	cat#4060, lot#23
Anti-vinculin antibody	Sigma	cat#V9131
Anti-alpha tubulin antibody	Sigma-Aldrich	cat#T5168, lot#115M482

**Transfection Reagents and Antibiotics**		
Lipofectamine 3000	Invitrogen by Thermo Fisher Scientific	cat#L3000150
Polyethylenimine (PEI)	Polysciences, Inc.	cat#23866-1, lot#690174
Polybrene	Sigma	cat#TR1003
Ampicillin	Fisher Bioreagnets	cat#BP1760-25, lot#185595
Puromycin	Fisher BioReagents	cat#58-58-2, lot#184968
Blasticidin	Calbiochem	cat#203350
**Experimental Models: Cell Lines**		
HEK293T	UNC Tissue Culture Facility	
T98G	Dr. Wenyi Wei (BIDMC)	
HeLa	Dr. Wenyi Wei (BIDMC)	
BPH1	Dr. Greg Wang (UNC)	
A673	Dr. Ian Davis (UNC)	
MHH-ES-1	Dr. Ian Davis (UNC)	
A549	Dr. Chad Pecot (UNC)	
H1299	Dr. Chad Pecot (UNC)	
H358	Dr. Chad Pecot (UNC)	
UMRC2	Dr. Qing Zhang (UTSW)	
UMRC6	Dr. Qing Zhang (UTSW)	
RCC4	Dr. Qing Zhang (UTSW)	
786-O	Dr. Qing Zhang (UTSW)	
A498	Dr. William Kim (UNC)	
Mouse vascular smooth muscle	Dr. Christopher Mack (UNC)	
Calu-3	UNC Tissue Culture Facility	
**Software**		
Origin7 (Microcal)	OriginLab Corporation	https://microcal-origin.joydownload.com/
SPSS Statistics	IBM Corporation	SPSS 11.5 Statistical Software
**Others**		
Rneasy Mini Kit	QIAGEN	cat# 74106
iScript™ Reverse Transcription Supermix for RT-qPCR	Bio-Rad	cat# 170-8891
PowerUp™ SYBR™ Green Master Mix	Appliedbiosystems by Thermo Fisher Scientific	cat# A25742, lot# 00718807
Protease Inhibitor Cocktail	Bimake	cat# B14012, lot# 411013

Phosphatase inhibitor cocktail A and B	Bimake	cat# B15001-A /B15001-B, lot# 510028
MG132	Calbiochem	cat#474790
D4476	Sigma	cat#D1944
Epiblastin A	Tocris	cat#6340
lenalidomide	Sigma	cat#SML2283
thalidomide	Cayman chemical	cat#14610
CC-122	Medchemexpress	cat#HY-100507
pomalidomide	Cayman chemical	cat#19877
Angiotensin II	Sigma	cat#A9525
Dimethyl sulfoxide	Fisher Chemical	cat#D128-1, lot#192803
ProLong™ Gold Antifade Mountant with DAPI	Invitrogen	cat# P36931, lot#1926936
SARS-CoV-2 S conditioned pseudovirus	Montana Molecular	cat#C1110G
CellTiter-Glo 2.0 Cell Viability Assay	Promega	cat#G9241
Anti-fade DAPI mounting solution	Invitrogen	cat#D1306
Steady-Glo Luciferase Assays System	Promega	cat#E2510
**Deposited Data**		
Original microscope images and uncropped western blot images can be found at the DOI URL:	N/A	

Colony formation assays

Indicated cells were seeded into 6-well plates (600 cells/well) and cultured in 37°C incubator with 5% CO_2 for ~14 days until formation of visible colonies. Colonies were washed with 1xPBS and fixed with 10% acetic acid/10% methanol for 30 min, stained with 0.4% crystal violet in 20% ethanol for 30 min and washed by tap water and air-dried. Colony numbers were manually counted. Three independent experiments were performed to generate the error bars.

Immunofluorescence microscopy

Cells were grown on glass coverslips for 24 hours and fixed with 4% formaldehyde in 1xPBS for 10 minutes at room temperature and permeabilized with 0.1% Triton X-100 in 1x PBS for 10 minutes. Coverslips were rinsed 3 x 5 minutes with 1x PBS and mounted onto slides using prolong gold anti-fade reagent containing 4,6-diamidino-2-phenylindole (DAPI) (Invitrogen cat#D1306). Pictures were taken using a Keyence BZ-X700 microscope.

SARS-CoV-2 S conditioned pseudovirus infection assays

~15,000 UMRC2 cells in 100 µL culture media were plated into each well with triplicates in 96-well plates. Indicated concentrations of lenalidomide were added to cell culture 8-12 hrs post cell seeding, followed by adding 50 µL or 25 µL SARS-CoV-2 S protein conditioned pseudoviruses (Montana Molecular C1110G) as indicated in figures. The viral titer is 2 x 10^{10} viral genes (VG) per milliliter (mL) from manufacturer's instructions. 24 hrs post-infection, cells were washed with sterile DPBS twice and stored in 100 µL DPBS for reading GFP signals using the BioTek Cytation 5 Cell Imaging reader.

Packaging of home-made SARS-CoV-2 S protein condition pseudoviruses and infection assays

SARS-CoV-2 spike-pseudotyped HIV was generated *via* co-transfection of HEK293T cells with HIV clone pNl4.3-luciferase (4.5 µg) and pCAG-nCoV-S-FLAG (0.5 µg) by Lipofectamine 3000 as per manufacturer's instructions. Spike-pseudotyped HIV viral containing culture supernatant was harvested 3-days post-transfection and stored in 1 mL aliquots at -80°C.

UMRC2 cells (1 x 10^5 cells/well) in 1 mL culture media were plated in 24-well plates and incubated overnight. Target cells were then pre-treated with 40 µM lenalidomide for 7 hrs. Cell medium was removed prior to spininoculation and each well received 500 µL viral containing culture supernatant and proceeded to spininoculation at 1200 x *g* for 2 hr at 25°C. Indicated target cell groups were treated with 40 µM lenalidomide immediately after centrifugal inoculation, 24 hr and 48 hr post centrifugal inoculation. Cells were lysed 72 hr post spininoculation with Reporter Lysis Buffer (Promega, WI, USA) and firefly luciferase activity was detected by luciferase assays (Promega, WI, USA).

Statistics

Differences between control and experimental conditions were evaluated by Student's *t* test or *One-way ANOVA*. These analyses were performed using the SPSS 11.5 Statistical Software and $p < 0.05$ was considered statistically significant.

DATA AND SOFTWARE AVAILABILITY

All data supporting the findings in this study are available from the corresponding author upon reasonable request.

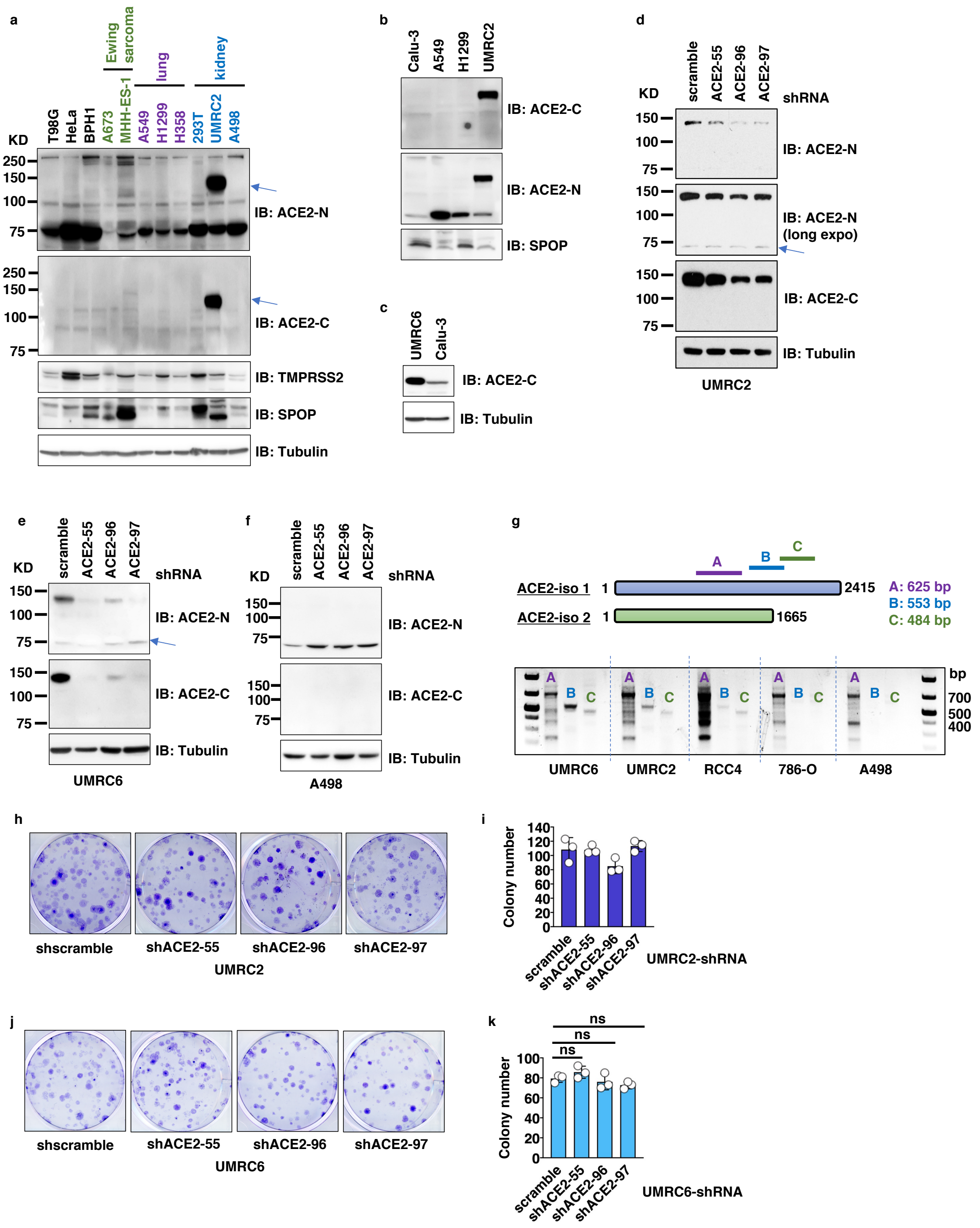

Figure. S1. Validation of ACE2 antibodies and cell lines used for this study.

(a-c) IB analysis of WCL derived from indicated cell lines. (**d-f**) IB analysis of indicated cell lines depleted of endogenous ACE2 by lenti-viral shRNAs. Cells were selected with 1 μg/ml puromycin for 72 hrs to eliminate non-infected cells before cell collection. (**g**) Top, a cartoon illustration of PRC primers designed to examine presence of ACE2 isoforms in indicated cell lines. Bottom, end-point PCR analyses for the presence of indicated PCR products. (**h-i**) Representative images (h)

and quantification (i) of colony formation assays using control and ACE2 depleted UMRC2 cells. 600 indicated cells were plated in 6-well plates with triplicates. (**j-k**) Representative images (j) and quantification (k) of colony formation assays using control and ACE2 depleted UMRC6 cells. 600 indicated cells were plated in 6-well plates with triplicates.

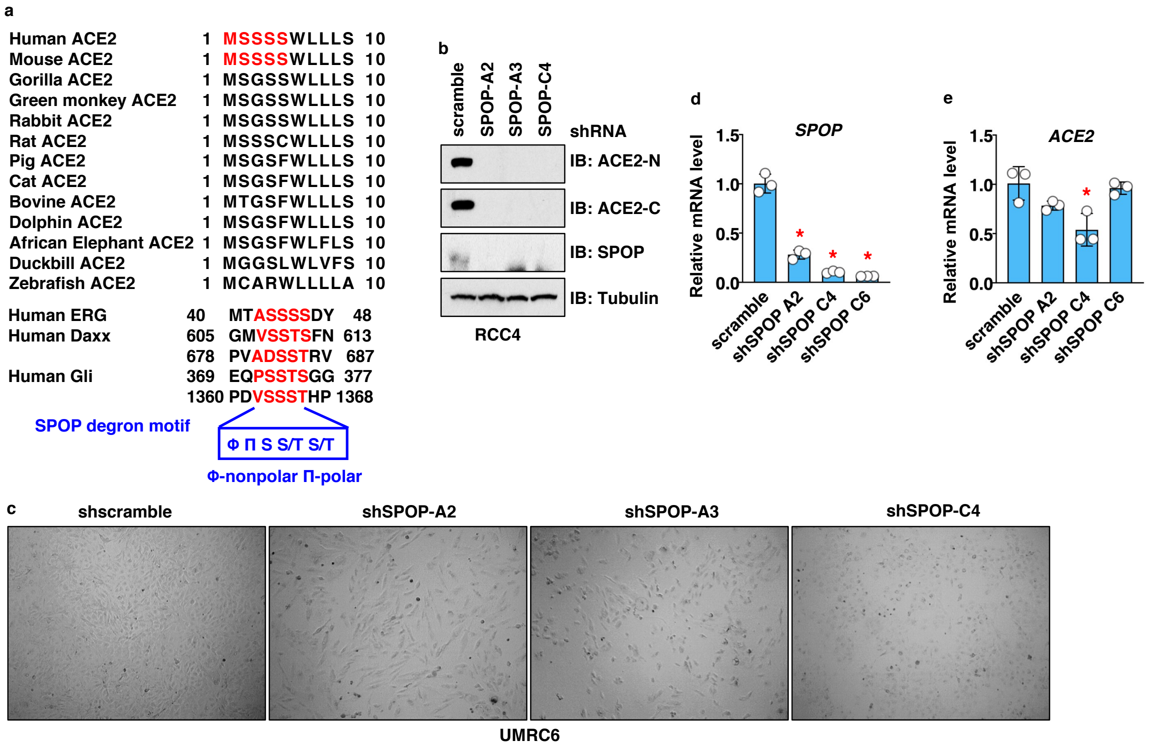

Figure. S2. SPOP deletion does not affect UMRC6 cell growth.

(**a**) Protein sequence alignment of SPOP degrons in ACE2 and other characterized SPOP substrates. (**b**) IB analyses of WCL derived from indicated RCC4 cells depleted of endogenous SPOP. (**c**) Representative images for cell morphology of indicated UMRC6 obtained from Fig. 2E. (**d-e**) RT-PCR analysis of SPOP (d) or ACE2 (e) mRNA levels in UMRC6 cells depleted of endogenous SPOP. * indicates $p<0.05$ from student's t-tests.

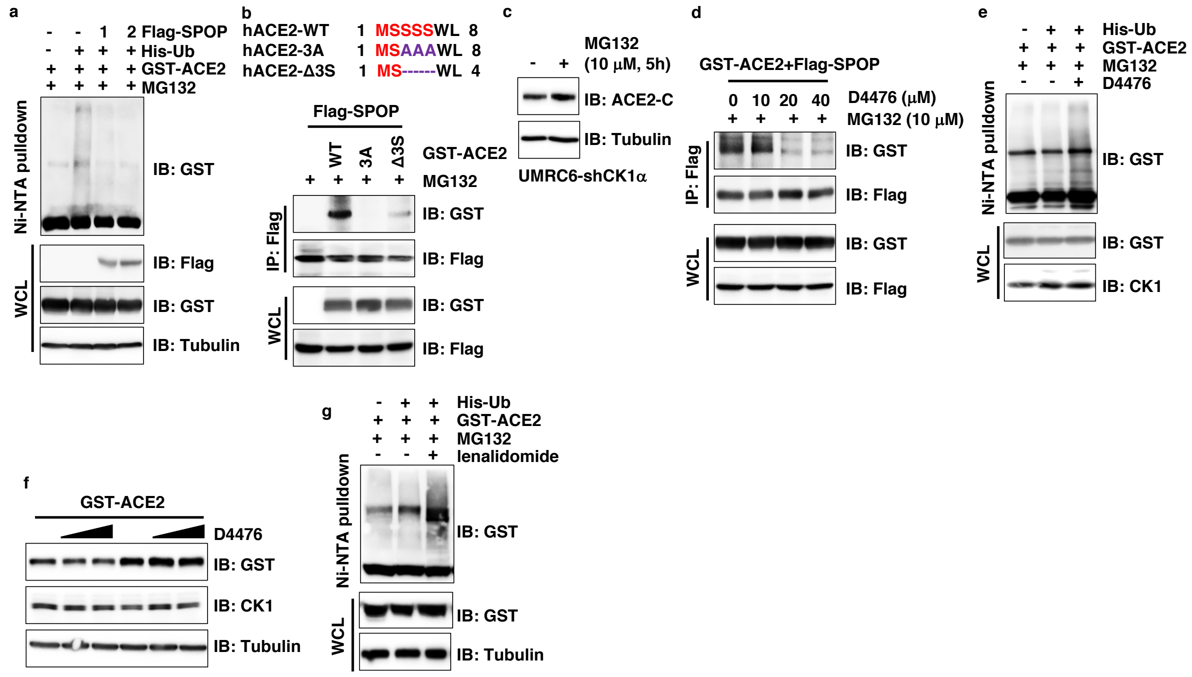

Figure. S3. CK1 phosphorylates ACE2 to prime ACE2 for SPOP binding and stabilization.

(**a**) IB analyses of WCL and Ni-NTA pulldowns derived from HEK293T cells transfected with indicated DNA constructs. 10 μM MG132 was added to cell culture 10 hrs prior to cell collection. (**b**) IB analyses of WCL and Flag-IPs derived from HEK293T cells transfected with indicated CMV-GST-ACE2 and Flag-SPOP. (**c**) IB analysis of WCL derived from UMRC6 cells depleted of endogenous CK1α isoform by lenti-viral shRNAs. Cells were treated with 10 μM MG132 for 5 hrs before cell collection. (**d**) IB analyses of WCL and Flag-IPs derived from HEK293T cells transfected with CMV-GST-ACE2 and Flag-SPOP. Indicated doses of D4476 and 10 μM MG132 was added to cell culture 10 hrs prior to cell collection. (**e**) IB analyses of WCL and Ni-NTA pulldowns derived from HEK293T cells transfected with indicated DNA constructs. 40 μM D4476 was added to cell culture 10 hrs prior to cell collection. (**f**) IB analysis of WCL derived from HEK293T cells transfected with indicated CMV-GST-ACE2 constructs. 10 or 40 μM D4476 was added to cell culture 10 hrs prior to cell collection. (**g**) IB analyses of WCL and Ni-NTA pulldowns derived from HEK293T cells transfected with indicated DNA constructs. 10 μM lenalidomide was added to cell culture 10 hrs prior to cell collection.

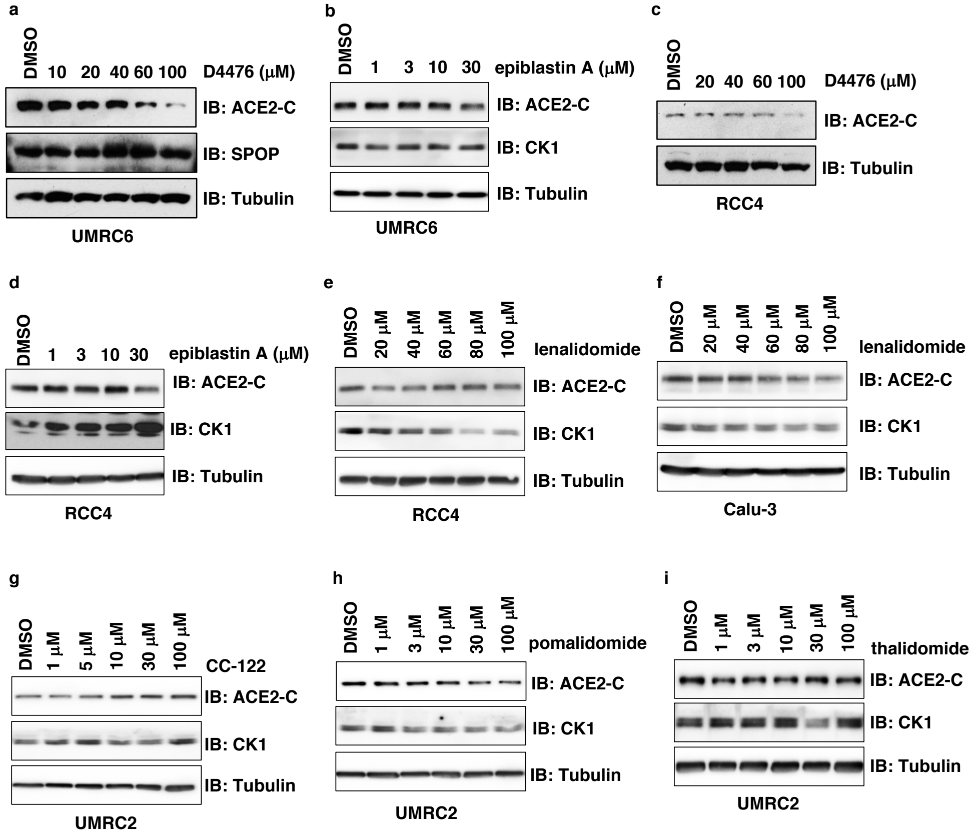

Figure. S4. CK1 inhibition leads to reduced ACE2 protein abundance in cells.

(**a-b**) IB analyses of WCL derived from UMRC6 cells treated with indicated doses of CK1 inhibitor D4476 or epiblastin A for 10 hrs. (**c-d**) IB analyses of WCL derived from UMRC6 cells treated with indicated doses of CK1 inhibitor D4476 or epiblastin A for 10 hrs. (**e-f**) IB analyses of WCL derived from RCC4 or Calu-3 cells treated with indicated doses of lenalidomide for 10 hrs. (**g-i**) IB analyses of WCL derived from UMRC2 cells treated with indicated doses of CC-122 (g), pomalidomide (h) or thalidomide (i) for 10 hrs.

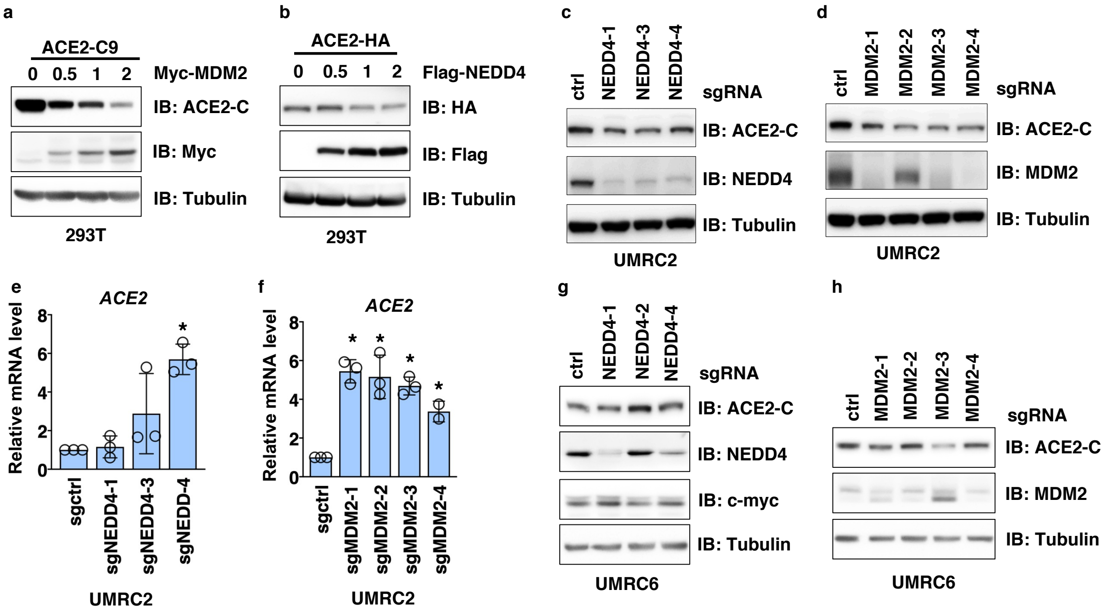

Figure. S5. Depletion of NEDD4 or MDM2 does not lead to ACE2 accumulation in UMRC2 and UMRC6 cells.

(**a-b**) IB analyses of WCL derived from HEK293T cells treated with indicated doses of Myc-MDM2 (a) or Flag-NEDD4 (b) with indicated ACE2 constructs. (**c-d**) IB analysis of UMRC2 cells depleted of endogenous NEDD4 or MDM2 by lenti-viral shRNAs. Cells were selected with 1 μg/ml puromycin for 72 hrs to eliminate non-infected cells before cell collection. (**e-f**) RT-PCR analyses of ACE2 mRNA levels in cells obtained in c and d. Error bars were calculated as mean+/-SD, n=3. * indicates $p < 0.05$ (one-way *ANOVA* test). (**g-h**) IB analysis of UMRC6 cells depleted of endogenous NEDD4 or MDM2 by lenti-viral shRNAs. Cells were selected with 1 μg/ml puromycin for 72 hrs to eliminate non-infected cells before cell collection.

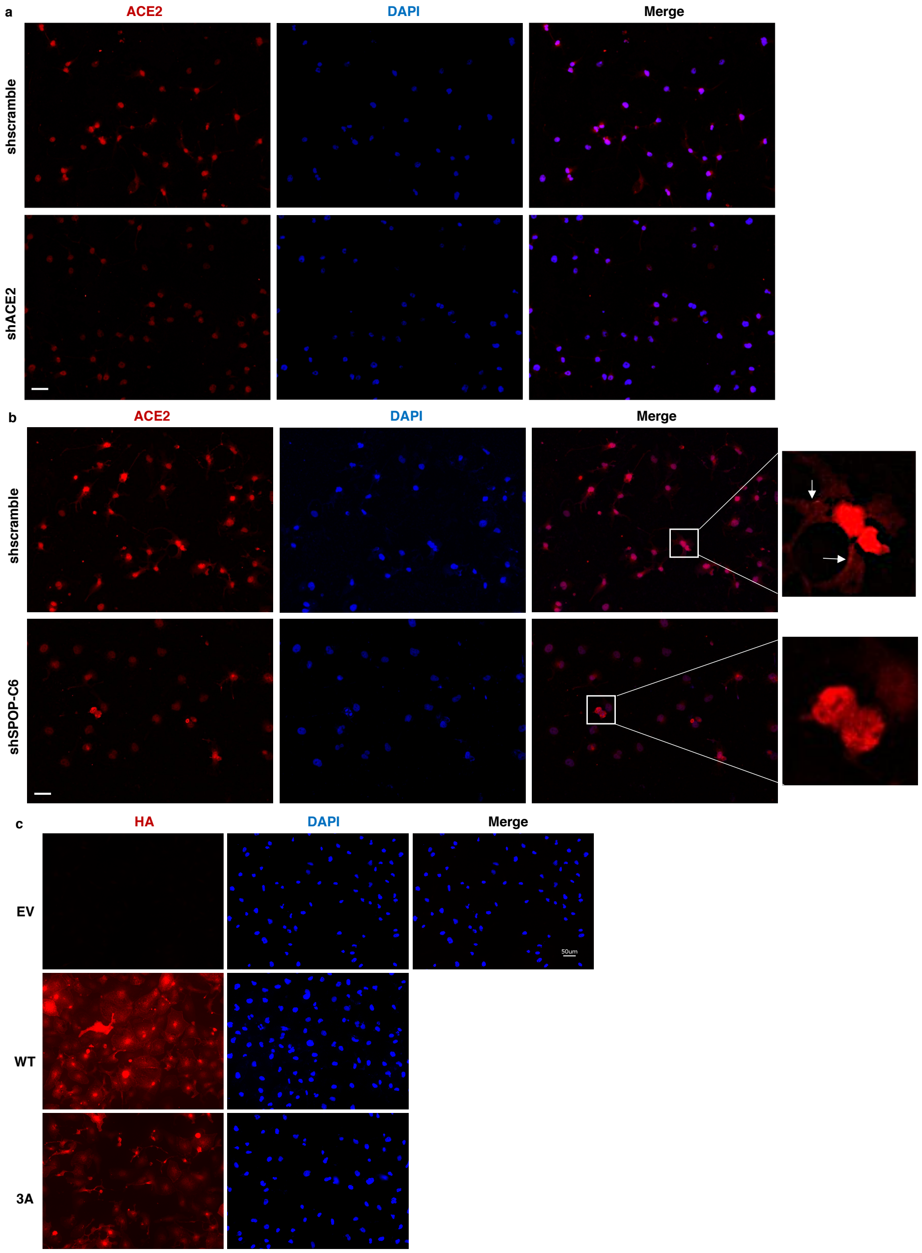

Figure. S6. Examination of ACE2 cellular localization.

(a) Representative immunofluorescent images indicating that ACE2-C antibody is suitable for detecting endogenous ACE2 proteins in IF. The bar indicates 50 µm. **(b)** Representative immunofluorescent images indicating that SPOP depletion reduces ACE2 proteins in both membrane and cytoplasm. The bar indicates 50 µm. **(c)** Representative immunofluorescent images indicating that 3A-ACE2 is deficient in localizing to plasma membrane. The bar indicates 50 µm.

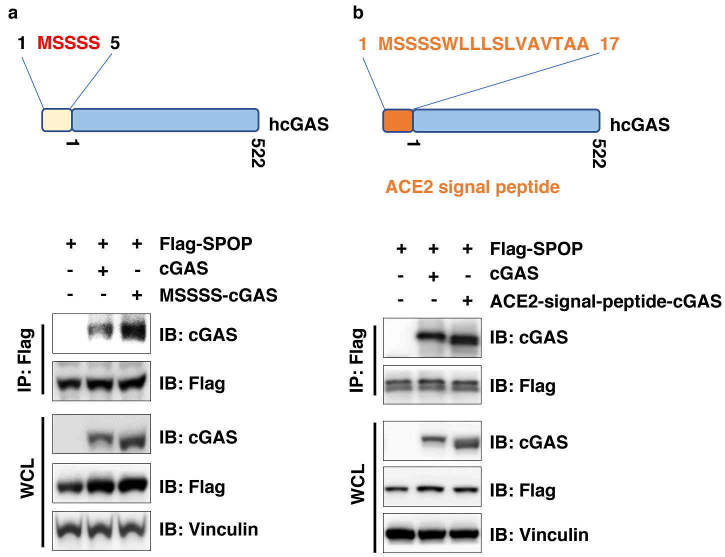

Figure. S7. Inserting the SPOP degron from ACE2 enhances cGAS binding to SPOP.

(**a-b**) IB analyses of WCL and Flag-IPs derived from HEK293T cells transfected with indicated DNA constructs. Inserted sequence of SPOP degron only (a) or the whole signal peptide (b) from ACE2 are indicated on top panels.

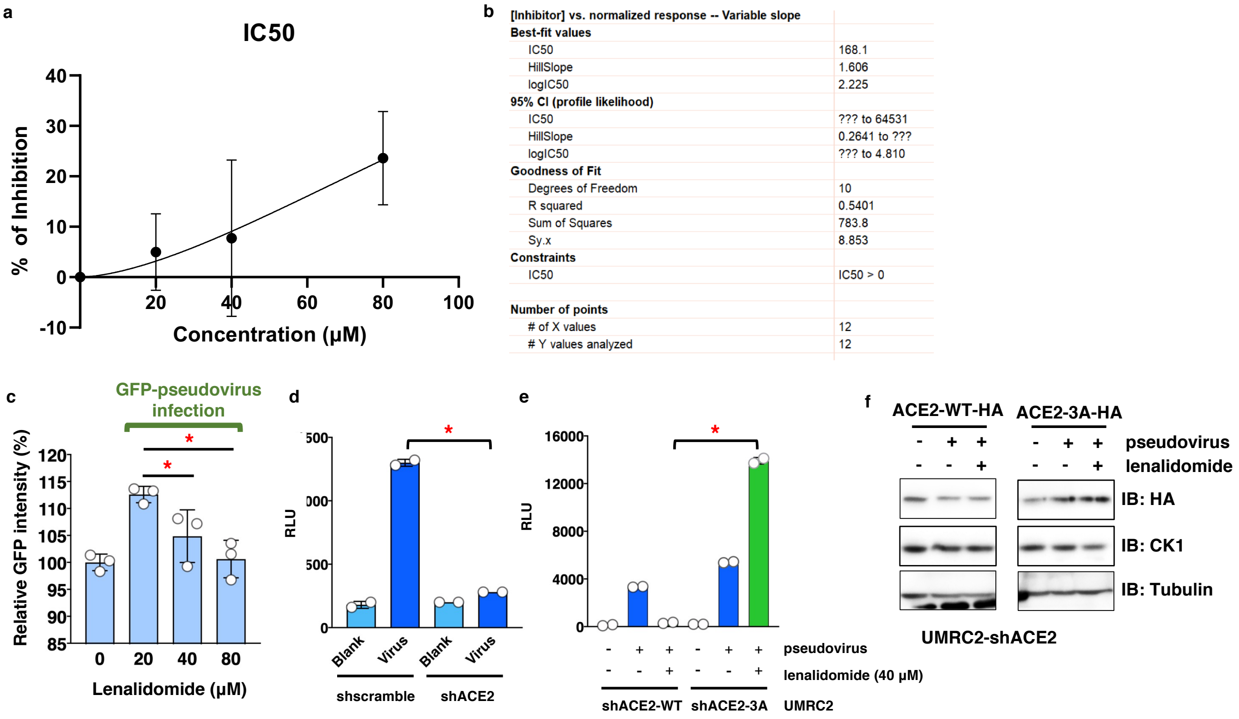

Figure. S8. 3A-ACE2 evades lenalidomide induced reduction in pseudoviral infection.

(**a-b**) A standard method to calculate IC50 for lenalidomide in Fig. 1x. (**c**) Treating UMRC2 cells simultaneously with 20, 40 or 80 μM lenalidomide together with GFP expressing SARS-CoV-2 S protein conditioned pseudoviruses reduced viral infection *in vitro*. GFP signals were measured 24 hrs post-infection. Error bars were calculated as mean+/-SD, n=3. *p < 0.05 (one-way *ANOVA* test). (**d-e**) Treating indicated UMRC2 cells with home-made pseudoviruses in ACE2 depleted cells (d) or reconstituted with WT or 3A-ACE2 in the presence of 40 μM lenalidomide pretreatment for 7 hrs. Error bars were calculated as mean+/-SD, n=2. * indicates $p < 0.05$ (one-way *ANOVA*). (**f**) IB analyses of WCL derived from indicated UMRC2 cells infected with 1.5 mL home-made S protein pseudotyped viruses for 24 hrs with a 7-hr pretreatment with 80 μM lenalidomide.

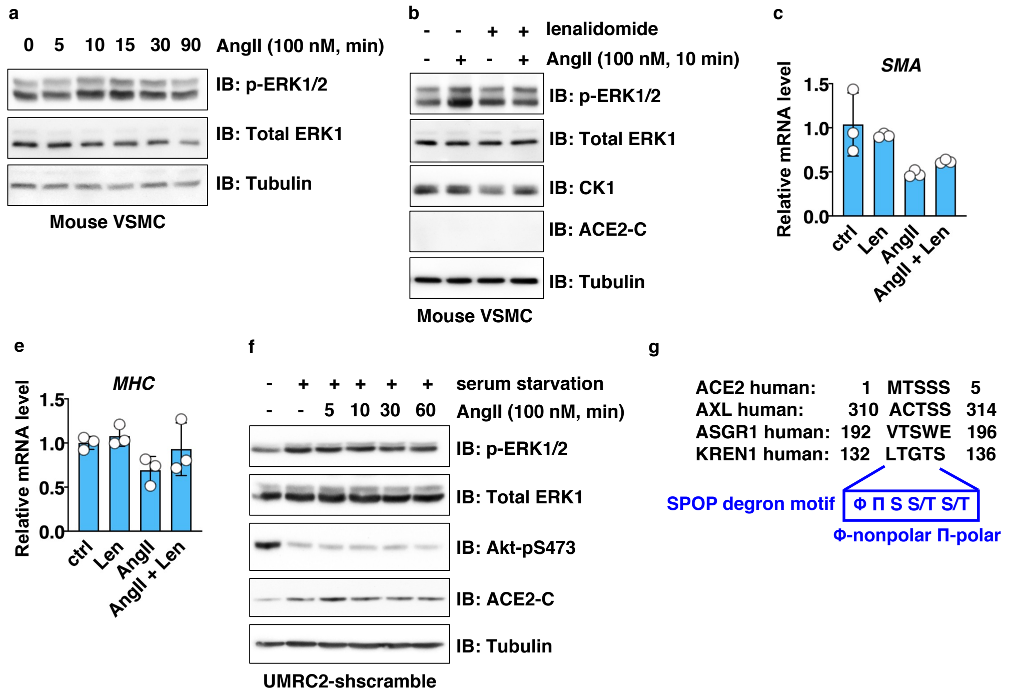

Figure. S9. Lenalidomide affects AngII effects in mouse VSMC cells.

(**a-b**) IB analyses of WCL derived from mouse VSMC (vascular smooth muscle cells) treated with indicated dose of AngII for indicated period after serum starvation for 24 hrs. Where indicated, 40 μM lenalidomide was added to culture 7 hrs prior to cell collection. (**c-e**) RT-PCR analyses of indicated mRNA level changes in VSMC cells with indicated treatment from (b). Len, lenalidomide. (**f**) IB analyses of WCL derived from UMRC2-shscramble cells treated 100 nM AngII for indicated period after serum starvation for 24 hrs. (**g**) Conservation of the SPOP degron in SARS-CoV-2 spike protein binding receptors including ACE2, AXL, ASGR1 and KREN1.
